# Stability of titania nanotube arrays in aqueous environment and the related factors

**DOI:** 10.1038/srep23065

**Published:** 2016-03-10

**Authors:** Can Cao, Jun Yan, Yumei Zhang, Lingzhou Zhao

**Affiliations:** 1 State key Laboratory of Military Stomatology, Department of Periodontology, School of Stomatology, The Fourth Military Medical University, Xi’an 710032, China; 2 State key Laboratory of Military Stomatology, Department of Prosthetic Dentistry, School of Stomatology, The Fourth Military Medical University, Xi’an 710032, China; 3 Department of Stomatology, General Hospital of Shenyang military region Command, China.

## Abstract

Titania nanotube arrays (NTAs) on titanium (Ti) fabricated by electrochemical anodization have attracted tremendous interest for diverse applications, of which most perform in aqueous environment or related to interaction with water. The NTAs are widely studied however the related factor of stability of NTAs when applied in such environment has rarely been concerned. We report that the annealed anatase NTAs are stable but the non-annealed amorphous NTAs are unstable to undergo specific structural change accompanied with a process of amorphous TiO_2_ dissolution and anatase TiO_2_ recrystallization. Quite unexpectedly, the non-annealed NTAs still show good stability without structural change in the cell culture media, possibly due to the presence of inorganics that may interfere with the TiO_2_ dissolution/redeposition process. The pH value of the aqueous environment is not a determinant factor for the structural change for non-annealed NTAs or not, while the temperature and the existence of F^−^ can accelerate the structural change process. F^−^ may play a very important role in the change process.

Over the past decade, titania nanotube arrays (NTAs) on titanium (Ti) fabricated by electrochemical anodization have attracted tremendous interest for diverse applications, of which most work in aqueous environment or related to the interaction with water, such as photocatalysis, photoelectrochemical water splitting, sensors, drug delivery and biological coatings^1^,^2^. There are researches focusing on the transformation of the as-grown amorphous NTAs to the crystalline TiO_2_ for specific applications^3^. It was reported that spontaneous phase and morphology transformation of as-formed NTAs occurred in pure water at room temperature^4^. A room-temperature spontaneous crystallization of amorphous titania to anatase in the absence of any solvent, additive or catalyst was also reported^5^. Lowering the transformation temperature can simplify the transformation conditions as well as avoid hindering the integration of TiO_2_ nanostructures with the thermally unstable polymeric substrates^6^,^7^,^8^,^9^. Normally, a stable structure is required for the normal function of a nanostructure once the optimal structure is determined. As many NTAs are used in the phase of amorphous in the environment of water, the finding of room-temperature spontaneous crystallization of as-formed NTAs initiates our concern on their stability.

As is known, the stability of a material is closely related to its composition and phase. NTAs are mainly composed of titania. It is noted that many factors in the fabrication and post-treatment of as-grown NTAs may potentially alter the composition and phase, such as electrolyte composition, annealing treatment and washing procedure, which are usually differential in various reports. In regard of the electrolyte composition, NTAs can be fabricated in F^−^ containing electrolytes with water solvent^10^,^11^ or some polar organic solvents such as ethylene glycol (EG)^12^,^13^, leading to obvious disparity in the nanotube form, diameter and length. The washing procedure for NTAs is not standardized but usually arbitrarily chosen by researchers. In some studies they were thoroughly cleaned ultrasonically^14^,^15^,^16^,^17^, but in some others just briefly cleaned^4^. The washing procedure will directly affect the F^−^ residue from the electrolytes. For some applications, annealing treatment was conducted to transform the as-formed amorphous NTAs into crystalline anatase, such as dye-sensitized solar cells^18^ and photocatalysis^19^, but for some other applications annealing treatment was not essential, such as drug delivery and biological coatings^20^. Whether and to what extent these factors influence the stability of NTAs in the aqueous environment or related to the interaction with water are largely unknown. In this study, the stability of NTAs fabricated in the electrolytes with water solvent or organic EG solvent, thoroughly washed or not, and annealed or not in the aqueous environments was observed (Fig. 1). The data hold great significance for the application of the NTAs in the aqueous environment or related to the interaction with water.

## Results and Discussion

Firstly, the morphology and phase shift of NTAs soaked in distilled water at 37 °C was inspected. Grossly, the as-anodized NTAs formed in EG electrolyte (Fig. S1) and water electrolyte (Fig. S2) both showed a dark brown appearance. The 3-hour annealing treatment at 450 °C changed their gross appearance while the washing procedure induced no discernible change. After 1-day water soaking, regardless of washing or not, the non-annealed NTAs fabricated in EG electrolyte decolorized. The gross appearance turned to be white, a characteristic of crystalline TiO_2_ (Fig. S1A,B). On contrary, the annealed counterparts showed no discernible gross appearance change after as long as 7-day water soaking (Fig. S1C,D). For the NTAs formed in water electrolyte, no obvious change in the gross appearance was witnessed after water soaking, regardless of washing and annealing or not (Fig. S2). As shown later, the non-annealed NTAs formed in water electrolyte also underwent a phase and morphological change after water soaking similar to those formed in EG electrolyte. The reason for the no gross appearance change shall be attributed to the fact that the NTA film formed in aqueous electrolyte is too thin to affect the optical appearance.

**Figure 2:**
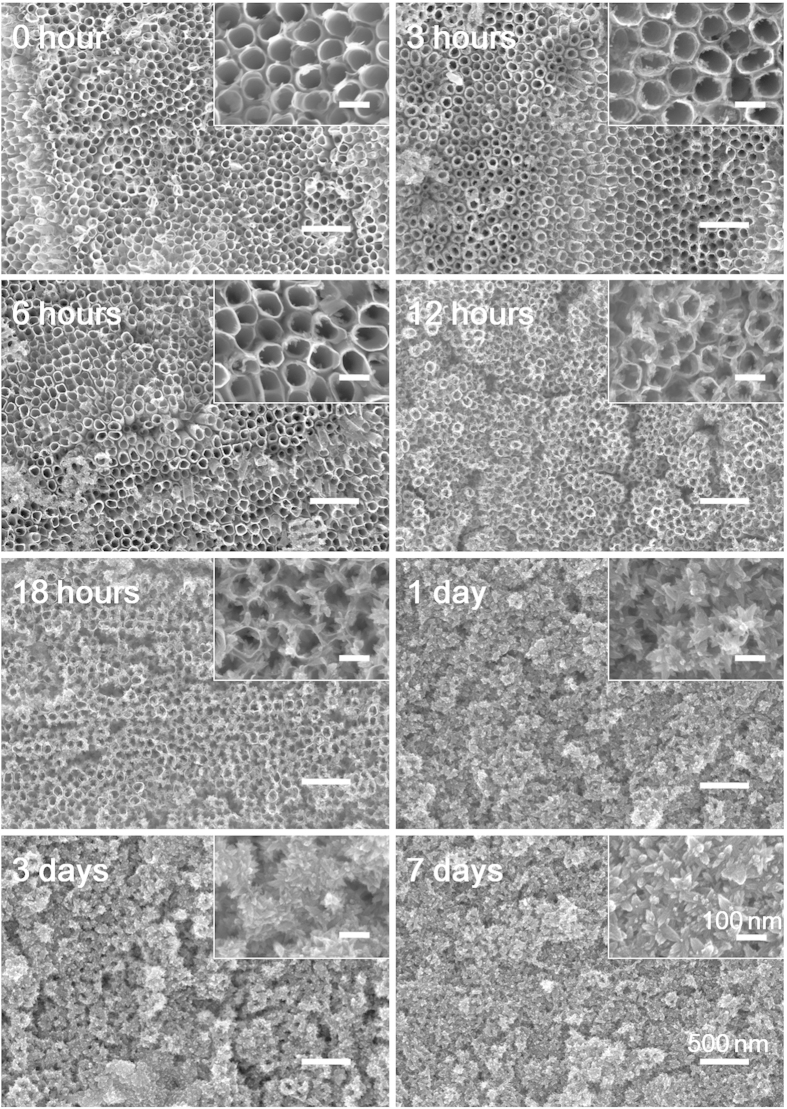
The microstructure of the non-annealed but washed NTAs fabricated in EG electrolyte before and after soaking in the deionized water for 7 days. Insets show the higher magnification images. The pH value of the solution is 7.53 ± 0.02.

The structure of NTAs was inspected by field emission scanning electron microscopy (FE-SEM). The NTAs formed in EG electrolyte had nanotubes of relatively regular form with even diameter of 80 nm and wall thickness of 10 nm (Fig. 2), while those formed in aqueous electrolyte had nanotubes of much irregular in form with an average diameter of 80 nm (Fig. S3). The annealing treatment or washing process did not influence the microstructure of NTAs (Figs S4 and S5). For the annealed NTAs there was no discernible microstructural change after water soaking (Fig. S4), but on contrary dramatic structural change was observed for the non-annealed NTAs regardless of the electrolyte type and washing or not (Figs 2, S3 and S5). After 3-hour water soaking, on the inner side wall of the non-annealed but washed nanotubes formed in EG electrolyte, there were abundant nano-prickles formed (Fig. 2). With the soaking time, the number and size of nano-prickles increased and meanwhile the nanotube wall became thinner. The growth rate of nano-prickles was a little faster on the unwashed counterparts (Fig. S5). After about half-day and one-day water soaking for the unwashed NTAs (Fig. S5) and the washed NTAs (Fig. 2), respectively, they were fully covered with nano-prickles to show a thorns-wreath like structure with a center pore diameter of 10 nm, which showed no more obvious structural change with further water soaking. A similar but slower structural change occurred for the non-annealed NTAs formed in aqueous electrolyte (Fig. S3).

**Figure 3:**
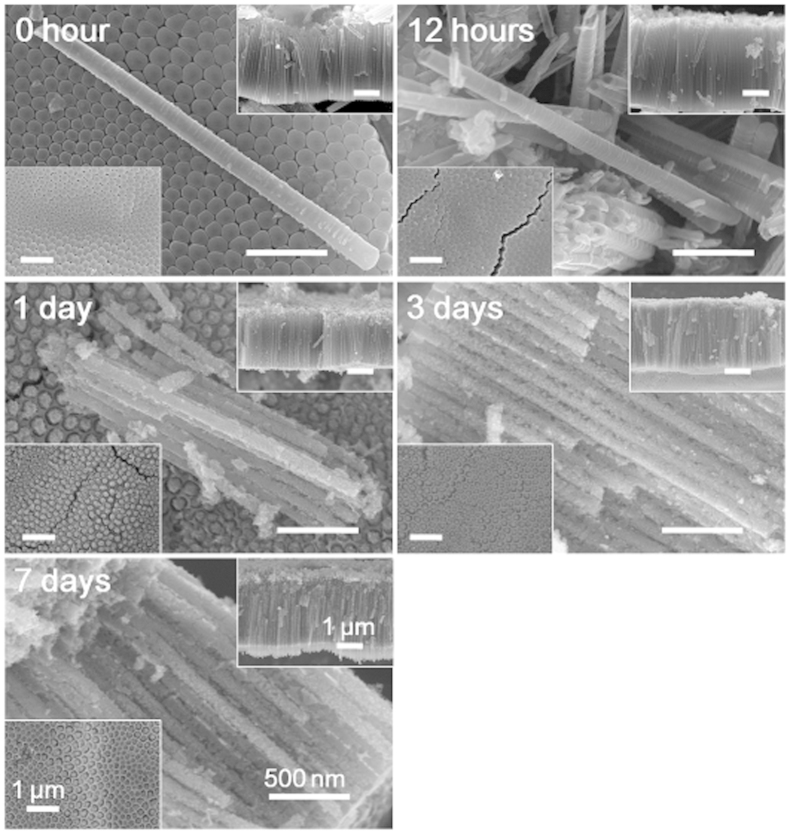
The side and bottom views of the non-annealed but washed NTAs fabricated in EG electrolyte before and after water soaking for 7 days. The upper right and lower left insets show the side and bottom views, respectively.

To inspect what happens in the deeper area of the non-annealed NTAs in water, the side and bottom of the NTA film were inspected after peeling off (Figs 3 and S6). After 1-day water soaking, there were nanoparticles formed that distributed evenly on the nanotube wall, regardless of being washed or not. The number and size of nanoparticles firstly increased with time from 1–3 days, and afterwards there was no more obvious change. On the bottom view, there were aggregates fulfilling the intertubular space after 1-day water soaking, indicating that the reaction happens along the entire length of the nanotubes. Such structural change shall lead to a more rigid connection among the adjacent nanotubes, as verified by the phenomenon that there were many single nanotubes for NTAs before and after 12-hour water soaking, while nearly no single nanotubes but bundles were found after more than 1-day water soaking.

**Figure 4:**
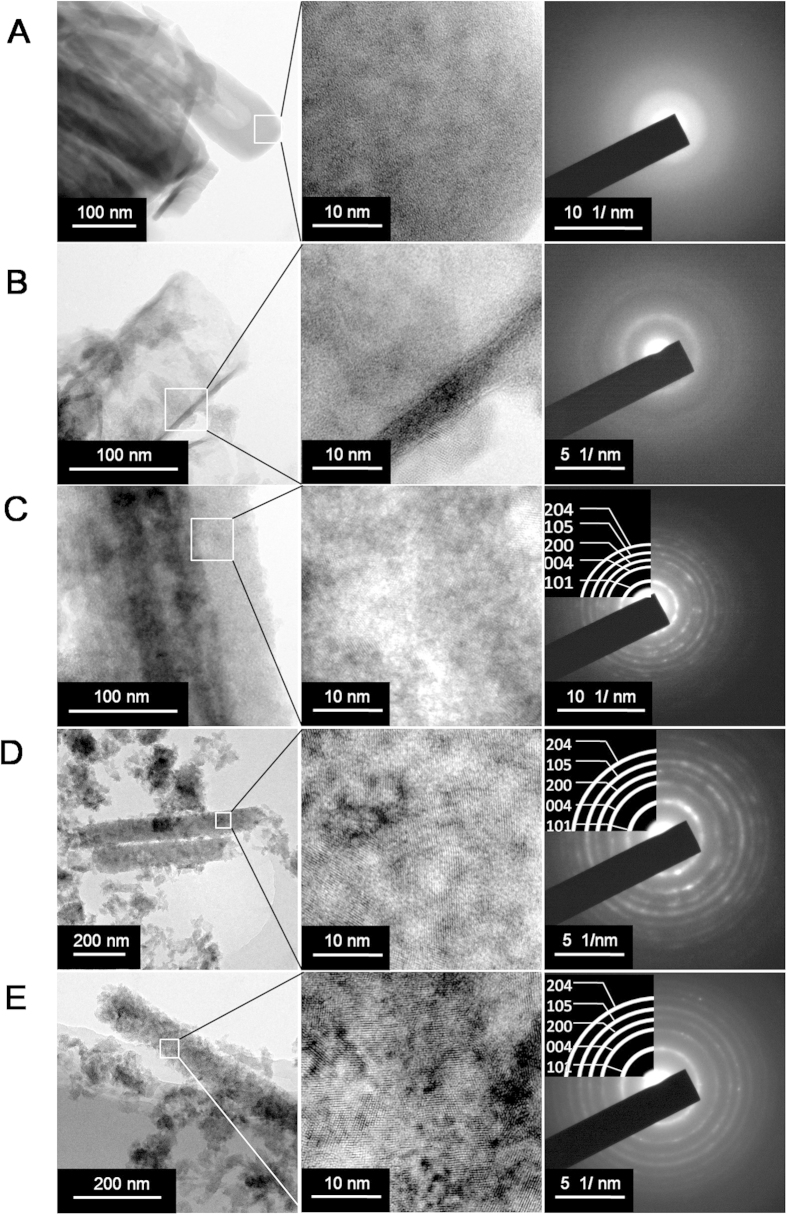
HRTEM and ED analyses on the non-annealed but washed NTAs fabricated in EG electrolyte (**A**) before and after water soaking of (**B**) 12 hours, (**C**) 1 day, (**D**) 3 days and (**E**) 7 days.

**Figure 5:**
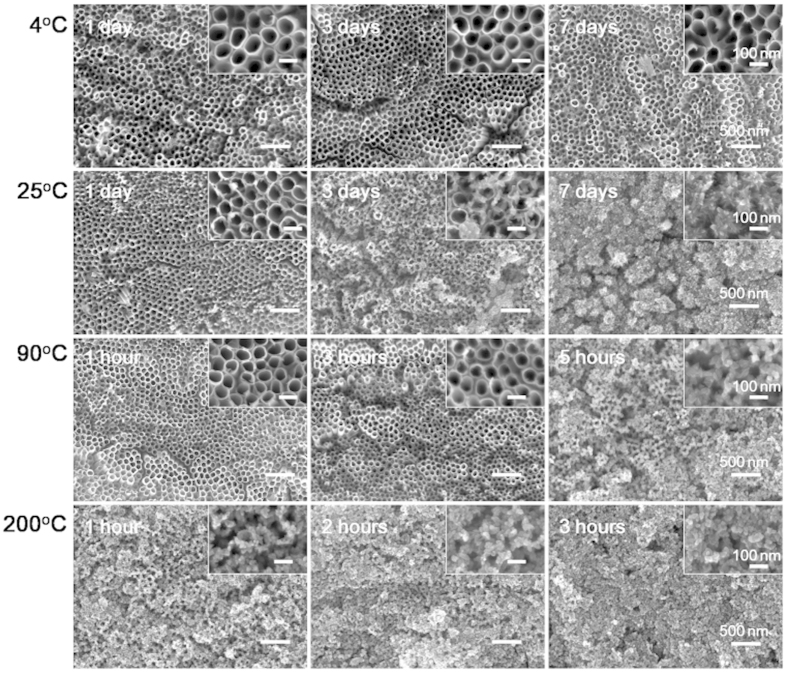
The structural change of the non-annealed but washed NTAs fabricated in EG electrolyte in the deionized water at different temperature. Insets show the higher magnification images.

To gain a deeper insight to the microstructural change of NTAs in water, high-resolution transmission electron microscopy (HRTEM) and electron diffraction (ED) analyses were carried out on the non-annealed but washed NTAs fabricated in EG electrolyte (Fig. 4). As displayed by HRTEM, before water soaking the nanotubes had a smooth tube wall composed of a homogeneous mazelike structure with no discernible lattice fringes, an indication of amorphous materials, which was further verified by the diffused ED pattern with no clear diffraction crystalline rings (Fig. 4A). After 12-hour water soaking (Fig. 4B), the nanotube wall was still clearly seen but became thinner. There were nanoparticles of 20–30 nm in size attaching to the nanotube wall. Since there is no extraneous Ti source, the nanoparticles shall be formed by self-sacrifice and recrystallization of the nanotube wall. The nanoparticles were in crystalline anatase phase, but the nanotube wall still retained amorphous. The distance of 0.35 nm between two neighbouring fringes can be clearly identified for the nanoparticles, corresponding to the d-spacing of (101) planes of anatase TiO_2_ 21. Nonetheless, the ED image shows unclear diffraction rings, possibly due to limited crystalline anatase nanoparticle formation. After 1-day water soaking (Fig. 3C), no more nanotubular structure could be observed, but instead nanowires composed of accumulated nanoparticles were witnessed. A set of well-defined rings of (101), (004), (200), (105) and (204) appeared in the ED pattern, indexed as anatase TiO_2_. The 3- and 7-day soaked NTAs (Fig. 4D,E) showed similar characteristics to the 1-day ones. The phase shift of NTAs in water was further confirmed by X-ray diffraction (XRD) (Fig. S7). For the unwashed NTAs, the anatase peaks appeared earlier and their intensity increased more quickly than the washed ones. These evidences demonstrate that accompanied with the structural change of the non-annealed NTAs in water is an amorphous TiO_2_ dissolution and anatase TiO_2_ recrystallization process. The water functions to induce the rearrangement of TiO_6_^2−^ octahedral units, resulting in the dramatically crystallization of anatase crystallites^22^. Our experimental data demonstrate that such transition accompanied with the unique structural change can occur in the facile condition of simple water soaking at 37 °C of body temperature, considering that the NTAs are widely studied in the biomedical field. The influence of temperature on the stability of non-annealed NTAs in water was also studied (Fig. 5). As expected, the structural change speed of non-annealed NTAs in water was positively related to the temperature.

**Figure 6:**
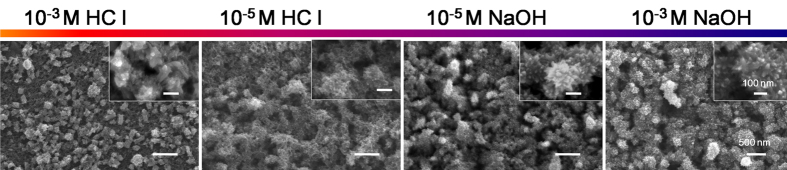
The structure of the non-annealed but washed NTAs fabricated in EG electrolyte in different concentrations of HCl and NaOH at 37 °C for 1 day. The pH values of the 10^−3^ M HCl, 10^−5^ M HCl, 10^−5^ M NaOH, 10^−3^ M NaOH solutions are 3.54 ± 0.02, 5.24 ± 0.03, 9.23 ± 0.03, 11.21 ± 0.02 respectively.

Then it is of interest to see whether such structural change for the non-annealed NTAs generally occurs in all aqueous environments, such as the biological environment. Taking the two commonly used cell culture media α minimum essential medium (α-MEM) supplemented with 15% fetal bovine serum (FBS) and phosphate buffered saline (PBS) as examples, it was surprisingly found that the structural change did not always occur (Fig. S8). The non-annealed NTAs showed good stability with no discernible structural and phase change in either α-MEM plus FBS or PBS, which shall be a good information for the applications of NTAs as biomedical coatings. Since the cell culture media are generally weak alkaline, we wonder if the pH value of the aqueous environment affects. Interestingly, obvious structural change happened in both the acidic and alkaline environments (Fig. 6), demonstrating that the pH value of the aqueous environment is not a determinant factor for the structural change for non-annealed NTAs or not. The good stability of the non-annealed NTAs in α-MEM plus FBS and PBS may be attributed to the presence of inorganics in these buffer solutions, which interfere with the thermodynamics of TiO_2_ dissolution and recrystallization thus impeding this process. Further study is necessitated to draw a conclusion.

**Figure 7:**
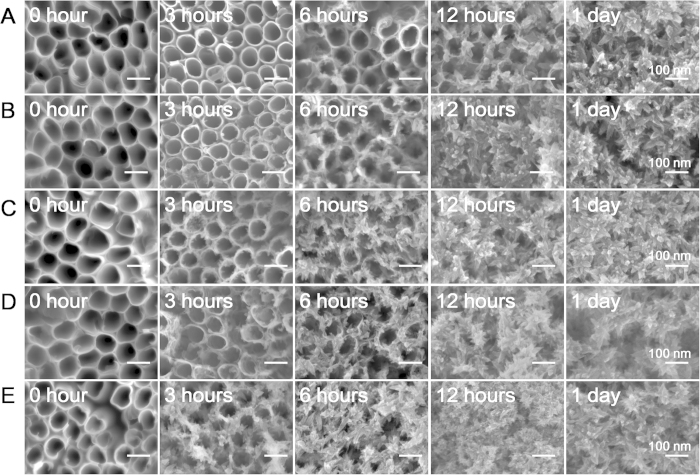
The structural change of the non-annealed but washed NTAs in (**A**) 0 mM, (**B**) 2 mM, (**C**) 5 mM, (**D**) 10 mM, (**E**) 20 mM of NH_4_F for different time at a relatively high magnification. The pH values of different solutions are (**A**) 7.53 ± 0.02, (**B**) 4.38 ± 0.03, (**C**) 4.24 ± 0.02, (**D**) 4.22 ± 0.01, (**E**) 4.18 ± 0.02, respectively.

The electrolyte for anodization contains fluoride, which gather around the anode of NTAs during anodization. After anodization, the regular washing procedure cannot remove the fluorine residue completely. Thus, X-ray photoelectron spectroscopy (XPS) revealed that there was fluorine distributing evenly along the nanotubes and the washing treatment could remove quite a part (Fig. S9), which may be the reason for the relatively slower structural change for the washed NTAs relative to the unwashed ones. To confirm this, the structural change of washed NTAs in different concentrations of NH_4_F was inspected (Fig. 7). The results showed that the existence of fluorine could indeed accelerate the structural change process and the changing rate is concentration closely related. Hence, our research suggested that the mechanism of the reaction is fluorine related. It is reported that TiO_2_ can react with fluoride generating titanium fluoride compound^23^ and the titanium fluoride compound can create anatase phase of TiO_2_ by hydrolysis^24^,^25^. In our study, the strengthened –OH bonds at 3126 cm^−1^ could been inspected (Fig. S12), which was also reported in previous studies^6^,^7^,^8^. The strengthened –OH bonds indicated the occurrence of the hydrolysis process. By this way, the presence of F- accelerate the amorphous TiO_2_ dissolution and anatase TiO_2_ recrystallization process.

## Materials and Methods

Pure Ti foils (BAOJI titanium industry) of 10 mm × 10 mm × 1 mm were polished by SiC sandpapers from 400–2000 grids to achieve a mirror-like surface, which were ultrasonically cleaned by acetone, ethanol, deionized water for 5 minutes each and dried in air. The NTAs were fabricated in the aqueous electrolyte containing 0.5 wt% HF at 20 V for 30 minutes or in the EG electrolyte containing 0.5 wt% NH_4_F, 5 vol% CH_3_OH and 5 vol% H_2_O at 40 V for 1 hour. Immediately after anodization, the NTAs were ultrasonically treated with acetone, ethanol and deionized water of 5 minutes each or not followed by drying with the nitrogen blow to give rise to the washed and unwashed NTA samples, respectively. The annealing treatment was conducted at 450 °C for 3 hours.

The NTA samples were immersed in deionized water, PBS and α-MEM of 1 ml for 3 hours to 7 days, which were dried by the nitrogen blow. The samples were characterized by FE-SEM (HITACHI S-4800), HRTEM (JEOL JEM-3010), XRD (Shimadzu-7000) with the target of Cu at 40.0 kV and 40.0 mA, EDS (HITACHI S-4800) for the fluoride distribution along the depth of the nanotubes, and XPS (ULVAC-Phi). The chemical structure of the samples was identified by the Fourier Transform InfraRed (FITR Vetex70). The pH values of the soaking solution were measured by a pH meter (Saturious FE20).

## Conclusions

In summary, in aqueous environment, the annealed anatase NTAs are stable but the non-annealed amorphous NTAs are unstable to undergo specific structural change accompanied with a process of amorphous TiO_2_ dissolution and anatase TiO_2_ recrystallization. Quite unexpectedly, the non-annealed NTAs still show good stability without structural change in the cell culture media, possibly due to the presence of inorganics that may interfere with the TiO_2_ dissolution/redeposition process. The pH value of the aqueous environment is not a determinant factor for the structural change for non-annealed NTAs or not, while the temperature and the existence of F^−^ can accelerate the structural change process.

## Additional Information

**How to cite this article**: Cao, C. *et al*. Stability of titania nanotube arrays in aqueous environment and the related factors. *Sci. Rep*. **6**, 23065; doi: 10.1038/srep23065 (2016).

## Supplementary Material

Supplementary Information

## Figures and Tables

**Figure 1:**
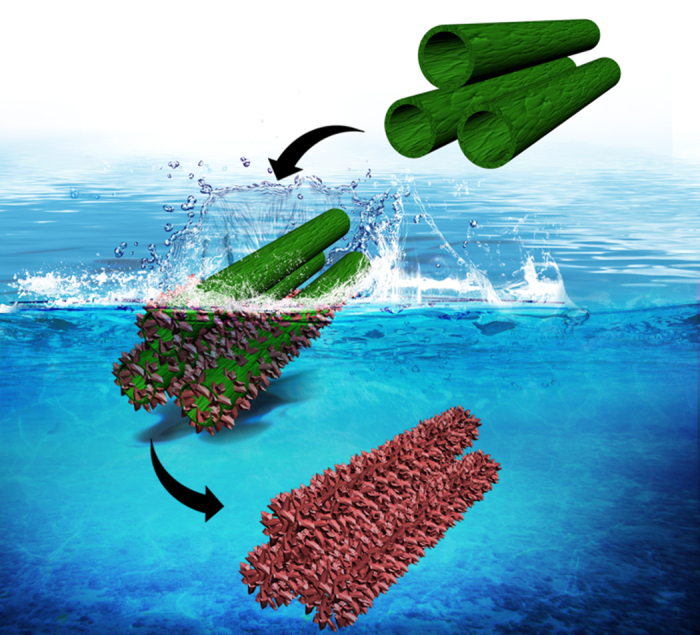
The amorphous titania nanotube arrays (NTAs) will undergo specific structural and phase change in aqueous environment except in cell culture media where they show good stability. The pH value of the aqueous environment does not obviously affect, while the temperature and the existence of F^−^ can accelerate the structural change process.
