# Supplementary material for: Stability of titania nanotube arrays in aqueous environment and the related factors
Source: Sci Rep. 2016 Mar 10;6:23065. doi: 10.1038/srep23065 (PMC4785341; doi:10.1038/srep23065)
Supplement: Supplementary Information [file srep23065-s1.doc]

Supporting Information

**Stability of titania nanotube arrays in aqueous environment and the related factors**

*Can Cao^b,c,d^, Jun Yan^b,d^, Yumei Zhang**^*b^ and Lingzhou Zhao^*a^*

*^a^ State key Laboratory of Military Stomatology, Department of Periodontology, School of Stomatology, The Fourth Military Medical University, Xi’an 710032, China*

*^b^ State key Laboratory of Military Stomatology, Department of Prosthetic Dentistry, School of Stomatology, The Fourth Military Medical University, Xi’an 710032, China*

*^c^ Department of Stomatology, General Hospital of Shenyang military command*

*^d^ Can Cao and Jun Yan contributed equally to this study*

*^*^Corresponding author:*

*State Key Laboratory of Military Stomatology, Department of Periodontology, School of Stomatology, The Fourth Military Medical University, No. 145 West Changle Road, Xi’an 710032, China. Dr. Lingzhou Zhao, E-mail:* [*zhaolingzhou1983@hotmail.com*](mailto:zhaolingzhou1983@hotmail.com)

*State Key Laboratory of Military Stomatology, Department of Prosthetic Dentistry, School of Stomatology, The Fourth Military Medical University, No. 145 West Changle Road, Xi’an 710032, China. Prof. Yumei Zhang; E-mail:* [*wqtzym@fmmu.edu.cn*](mailto:wqtzym@fmmu.edu.cn)


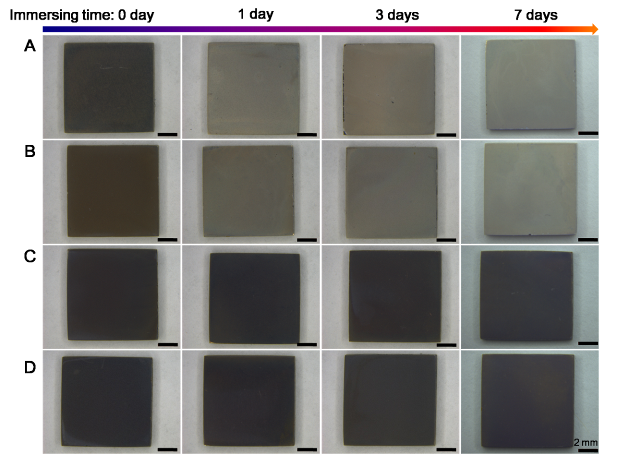


**Figure S1** Gross appearance of the NTAs on Ti fabricated in the EG electrolyte before and after immersion in the distilled water: (A) unwashed and non-annealed, (B) washed but non-annealed, (C) unwashed but annealed, (D) washed and annealed. The 4 rows from left to right show the NTAs before immersion and after 1, 3 and 7 days of immersion in the distilled water. Scale bars indicate 2 mm.


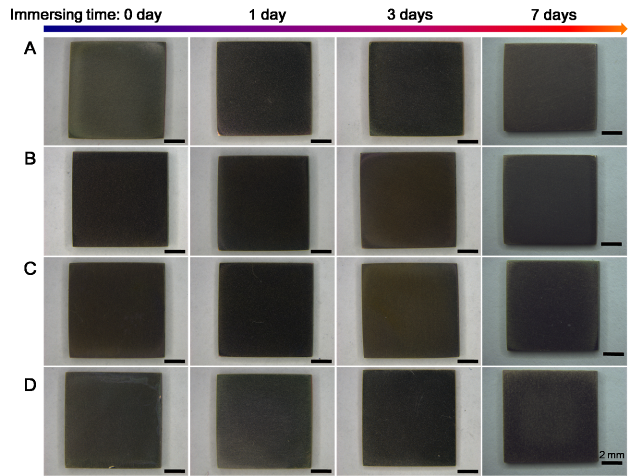


**Figure S2** Gross appearance of the NTAs on Ti fabricated in the aqueous electrolyte before and after immersion in the distilled water: (A) unwashed and non-annealed, (B) washed but non-annealed, (C) unwashed but annealed, (D) washed and annealed. The 4 rows from left to right show the NTAs before immersion and after 1, 3 and 7 days of immersion in the distilled water. Scale bars indicate 2 mm.


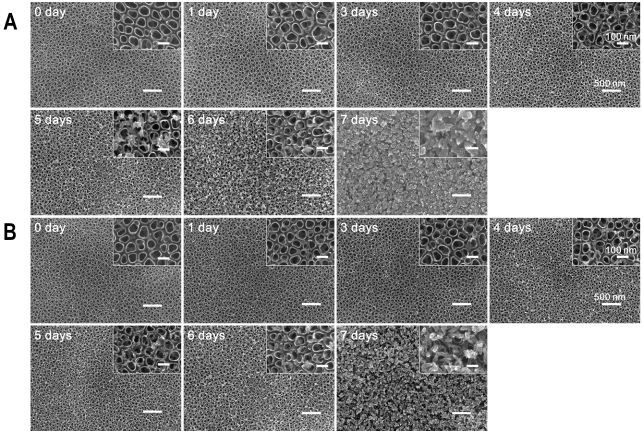
**Figure S3** The microstructure of the non-annealed NTAs fabricated in the aqueous electrolyte before and after immersion in the distilled water for 7 days: (A) the unwashed NTAs, (B) the washed NTAs. Insets show the higher magnification SEM images.


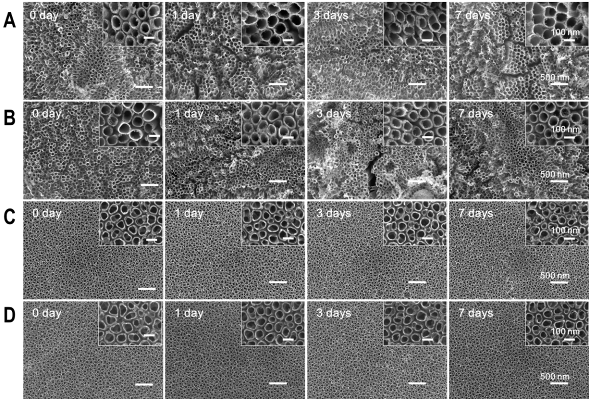

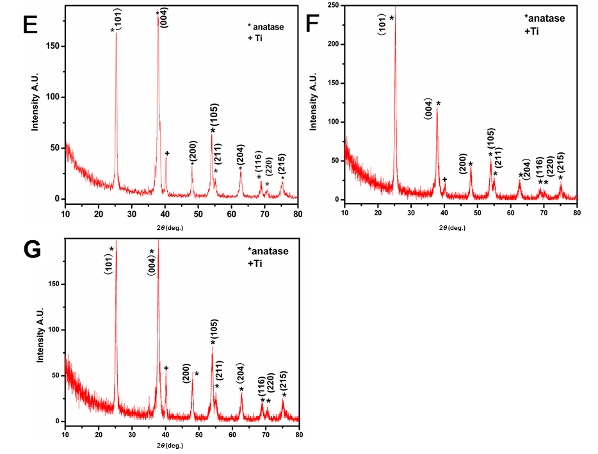


**Figure S4** The microstructure of the annealed NTAs on Ti before and after immersion in the distilled water for 7 days: (A) the NTAs fabricated in the EG electrolyte without washing, (B) the NTAs fabricated in the EG electrolyte with washing, (C) the NTAs fabricated in the aqueous electrolyte without washing, (D) the NTAs fabricated in the aqueous electrolyte with washing. The insets show the higher magnification SEM images. (E) The NTAs annealed at 450^o^C were mainly composed of anatase TiO_2_, as indicated by a set of strong XRD peaks at 2*θ*= 25.21, 37.81, 47.71, 54.21, 62.51, 70.64 and 77.56 corresponding to the (101), (004), (200), (105), (204), (220) and (215) planes of anatase TiO_2_, respectively. The annealed NTAs showed no phase change after water soaking for 3 days (F) and 7 days (G), in consistent with its structure stability.

**
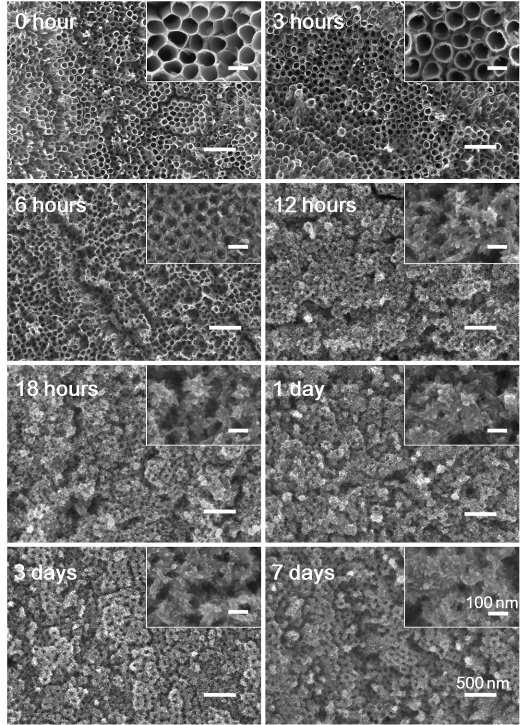
**

**Figure S5** The microstructure of the non-annealed and unwashed NTAs fabricated in the EG electrolyte before and after soaking in the distilled water for 7 days. Insets show the higher magnification SEM images.


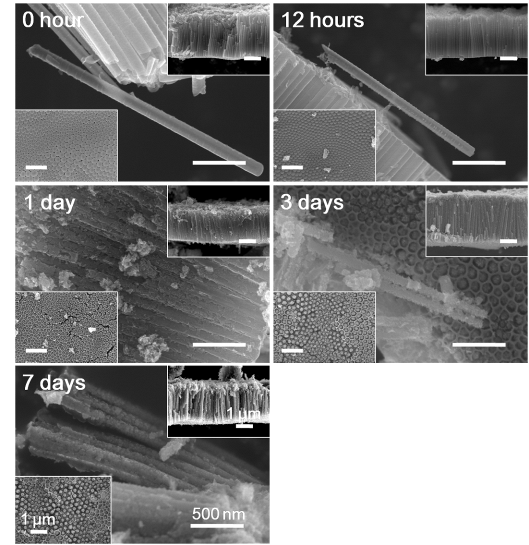


**Figure S6** The side and bottom views of the non-annealed and unwashed NTAs fabricated in the EG electrolyte before and after soaking in the distilled water for 7 days. The upper right corner insets show the integral cross-sectional topography. The lower left corner insets show the bottom view of the NTAs.


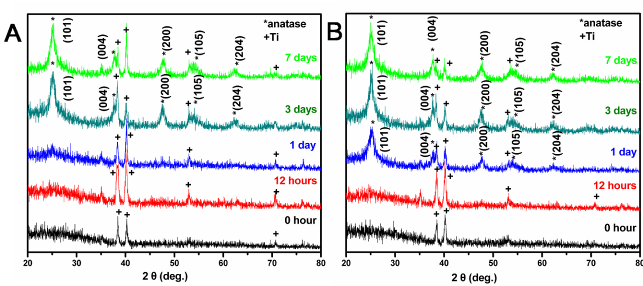


**Figure S7** XRD spectra of NTAs of (A) the non-annealed but washed fabricated, (B) the non-annealed and unwashed fabricated in the EG electrolyte before and after water soaking of 7 days. Both NTAs show amorphous phase and a time-dependent phase transfer from amorphous to anatase can be observed.


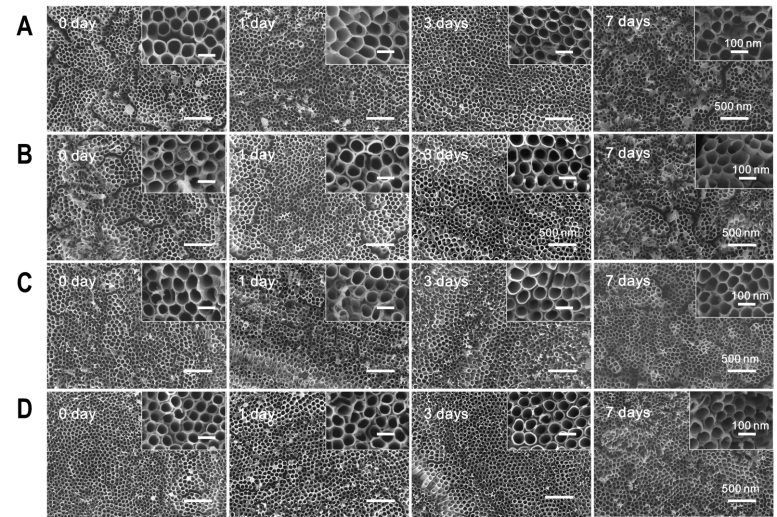

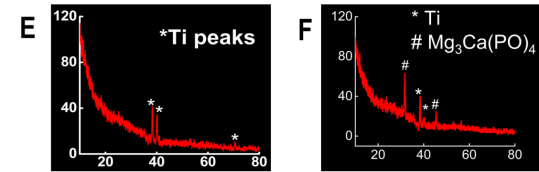


F**igure S8** The microstructure of the non-annealed NTAs fabricated in the EG electrolyte after immersion in the α-MEM plus serum or PBS for 7 days: (A) the unwashed NTAs soaked in the α-MEM plus serum, (B) the washed NTAs soaked in the α-MEM plus serum, (C) the unwashed NTAs soaked in the PBS, (D) the washed NTAs soaked in the PBS. The insets show the higher magnification SEM images. (E) and (F) show the representative XRD phases of the unwashed NTAs soaked in α-MEM plus serum and PBS for 7 days, respectively. The pH values of PBS solution and α-MEM plus serum are 7.20±0.02 and 7.60±0.02.


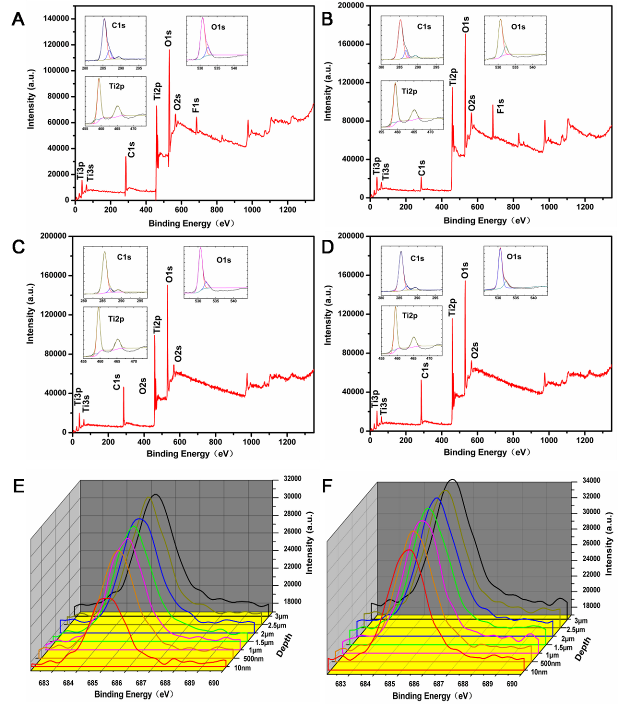


**Figure S9** The XPS spectra of NTAs fabricated in the EG electrolyte of (A) non-annealed but washed, (B) non-annealed and unwashed, (C) annealed and washed, (D) annealed but unwashed. The high resolution spectra of F1s at different depths of (A) non-annealed but washed NTAs, (B) non-annealed and unwashed NTAs is shown in (E) and (F) respectively. The inset images of (A), (B), (C), (D) show high-resolution spectra of O1s, Ti2p and C1s.


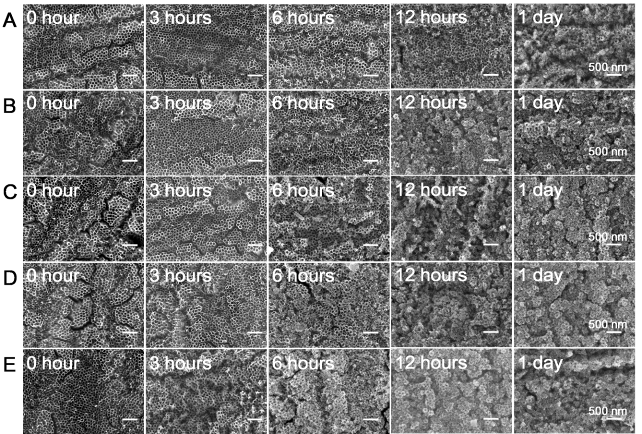


**Figure S10** The structural change of the non-annealed but washed NTAs soaked in (A) 0 mM, (B) 2 mM, (C) 5 mM, (D) 10 mM, (E) 20 mM of NH_4_F solution for different durations. The SEM images have a relatively lower magnification than those in Fig. 7 to show the panoramic view. The pH values of different solution are (A) 7.53±0.02, (B) 4.38±0.03, (C) 4.24±0.02, (D) 4.22±0.01, (E) 4.18±0.02 repsectively.


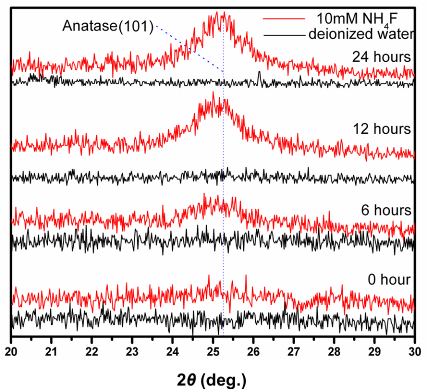


**Figure S11** The XRD spectra at the strongest peak of 2*θ* = 25.21 for the non-annealed but washed NTAs immersed in the 10 mM NH_4_F solution or the deionized water for different durations. The pH value of 10 mM NH_4_F solution is 4.22±0.01.

**
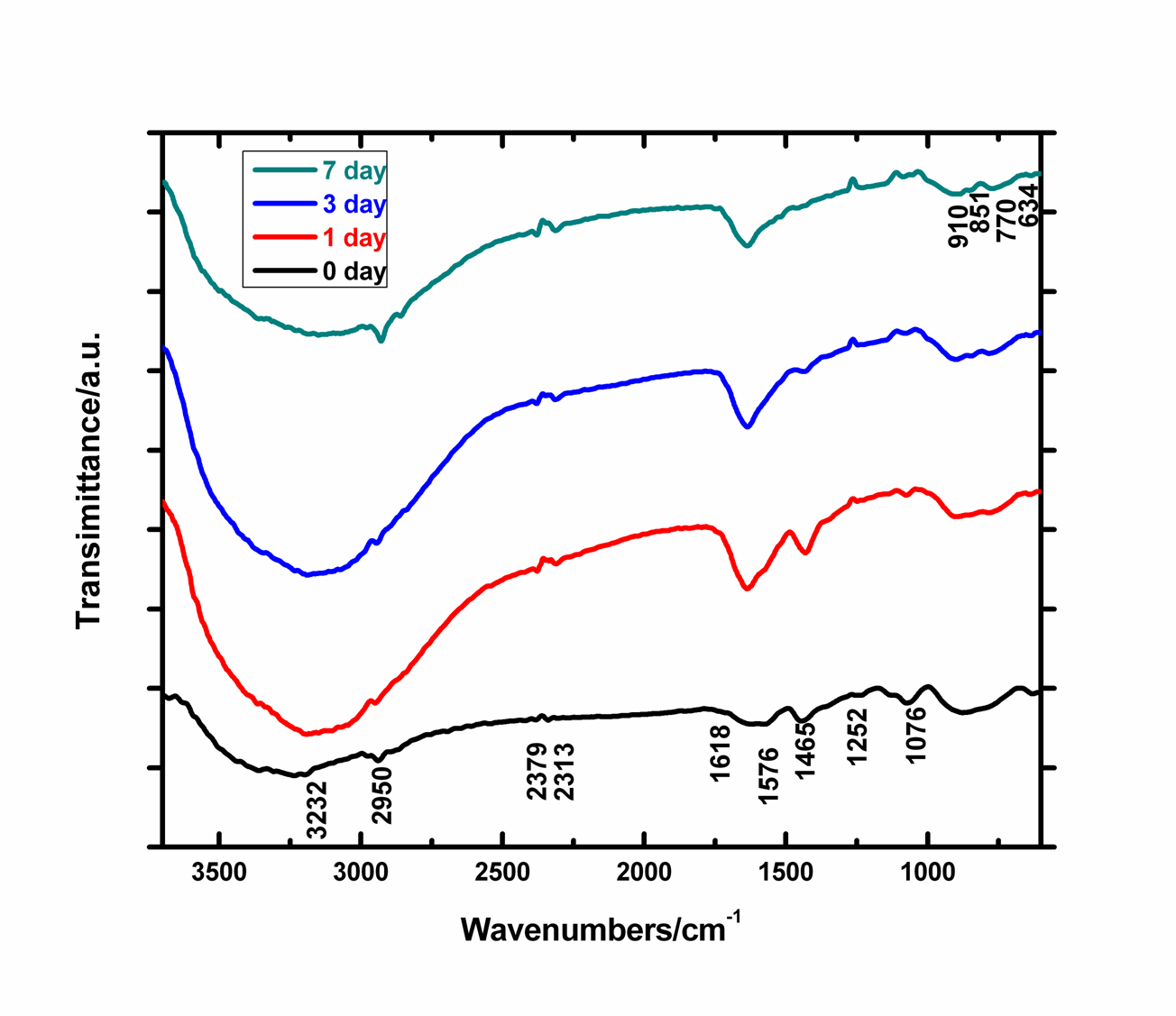
**

**Figure S12** The FTIR spectra of NTAs, and of the NTAs after immersed in water for different time.
